# Evaluation of orally administered *Megasphaera elsdenii* in steer calves abruptly transitioned from a receiving diet with 4% dietary starch to a growing diet with 38% dietary starch

**DOI:** 10.1093/tas/txae113

**Published:** 2024-07-24

**Authors:** Forest L Francis, Warren C Rusche, Zachary K Smith

**Affiliations:** Department of Animal Science, South Dakota State University, Brookings, SD 57007, USA; Department of Animal Science, South Dakota State University, Brookings, SD 57007, USA; Department of Animal Science, South Dakota State University, Brookings, SD 57007, USA

**Keywords:** backgrounding, cattle, diet transition, direct-fed microbial

## Abstract

The objective of this experiment was to evaluate the effects of orally administered *Megasphaera elsdenii* NCIMB 41125 as a microbial supplement in steers abruptly transitioned from a receiving diet with 4% dietary starch (dry matter [DM] basis) to a growing diet with 38% dietary starch (DM basis). Steers (*n* = 192; initial shrunk body weight [SBW] = 309 ± 20.6 kg) were assigned to microbial supplement treatment in a randomized complete block design. Treatments were control (CON): no microbial supplement prior to diet transition, and (DFM): microbial supplement orally administered prior to diet transition (20 mL of microbial supplement [Lactipro NXT, Axiota Animal Health, Fort Collins, CO] containing 1 × 10^10^ colony forming units *Megasphaera elsdenii* NCIMB 41125). Steers were sourced from a previously conducted 49 d feedlot receiving period experiment and abruptly transitioned from a receiving diet including soybean hulls and wheatlage containing 4% dietary starch (DM basis) to a growing diet including high-moisture ear corn, dry-rolled corn, and wheatlage containing 38% dietary starch (DM basis). Diets were switched on an equal DM intake basis to achieve the abrupt change and steers were fed the 38% starch diet for 49 d until experiment completion. Prior to experiment initiation, steers (*n* = 72; *n* = 3/pen) were fitted with wireless rumination tags to track daily activity and rumination time. No differences (*P* ≥ 0.20) were observed between treatments for final SBW, average daily gain, DM intake, feed efficiency, calculated net energy (NE) for maintenance and gain, or observed-to-expected ratio of NE for maintenance and gain. Additionally, no treatment × day or treatment differences (*P* ≥ 0.12) were observed for activity or rumination measures. Minutes ruminating and active both differed (*P* < 0.01) for the main effect of day. Compared to non-supplemented steers, oral administration of *Megasphaera elsdenii* NCIMB 41125 did not improve growth performance or efficiency of dietary NE utilization in steers transitioned from a receiving diet containing 4% starch (DM basis) to a growing diet containing 38% starch (DM basis).

## Teaser

The response of oral administration of *Megasphaera elsdenii* NCIMB 41125 at the time of abrupt change from a diet containing low starch content (4%) to higher starch content (38%) did not improve growth performance or efficiency of dietary net energy utilization.

## Introduction

In the United States, cattle fed in confinement are primarily fed diets with high levels of cereal grains. The main constituent of cereal grains is starch, which makes up between 60% and 80% of the grains weight (Huntington, 1997). As increasing starch is introduced into the diet of ruminants, populations (10^10^ cells/g) of amylolytic bacteria including *Streptococcus, Ruminobacter, Bacteroides, Butyrivibrio, Eubacterium, Selenomonas, Succinivibrio, Succinimonas,* and *Lactobacillus* increase (Kotarski et al., 1992). In grain-fed animals proportions of amylolytic bacteria can be as high as 90% to 95% of total culturable bacteria (Nagaraja and Lechtenberg, 2007). Of these amylolytic bacteria, *Selenomonas ruminantium, Streptococcus bovis, and Lactobacillus* sp. *S ruminantium* constitute between 22 to 51% percent of total culturable bacteria in grain-fed cattle and contribute to rapid accumulation of DL-lactic acid and VFAs which may result in acidotic conditions in the rumen (Nagaraja and Lechtenberg, 2007). Ruminal acidosis in feedlot cattle is characterized by the accumulation of acids that can damage ruminal and intestinal walls, decrease the pH of blood, and lead to dehydration (Owens et al., 1998). Additionally, as ruminal acidosis becomes chronic, symptoms of laminitis, polioencephalomalacia, and liver abscess can occur (Owens et al., 1998).

While there are numerous nutritional factors, management strategies, and feed additives that can be utilized to control acidotic conditions in the rumen, there is a normal microflora of lactate utilizing bacteria in the rumen that can survive and remain active in acidotic conditions. Under acidotic conditions caused by d and l-lactic acid buildup, *Megasphaera elsdenii* is the predominant ruminal lactate-utilizing organism (Nagaraja and Lechtenberg, 2007). *Megasphaera elsdenii* NCIMB 41125 (Lactipro; Axiota Animal Health, Fort Collins, CO) is a commercial patented strain that displays a high growth rate (up to 0.938/h) and biomass output (0.39 g [L/h]; Meissner et al., 2010). Additionally, this strain metabolizes lactate, produces fermentation end products well below 5.5 pH, and is unaffected by ionophores, most in-feed anthelmintics, and antibiotics (Meissner et al., 2010). Thus, this strain was selected as a superior candidate for use as a direct-fed microbial for prevention and control of lactic acidosis. The use of commercialized *Megasphaera elsdenii* NCIMB 41125 during in vitro and in vivo experiments has exhibited positive effects in the control of acidosis including increased lactate utilization, increased butyrate production, and decreased time spent and the area below 5.6 pH (Horn et al., 2009; Leeuw et al., 2016; Mazon et al., 2020).

Steers transitioned from an all-roughage diet to a high-concentrate diet and dosed with *Megasphaera elsdenii* NCIMB 41125 exhibited greater carcass-adjusted average daily gain (ADG), reduced prevalence of liver abscesses, and increased hot carcass weight compared to placebo treated controls (Drouillard et al., 2012). Early weaned calves transitioned to a blend of a 35% roughage growing and 10% roughage finishing diet and dosed with *Megasphaera elsdenii* NCIMB 41125 had improved ADG, and feed conversion (G:F) during days 1 to 42 of the trial and exhibited increased cumulative ADG over the entire feeding period compared to calves consuming a 35% roughage growing diet and calves consuming growing/finishing diet blend administered no *Megasphaera elsdenii* NCIMB 41125 (DeClerck et al., 2020). Holstein calves administered a dose of *Megasphaera elsdenii* NCIMB 41125 at 14 d old exhibited increased starter diet dry matter (DM) intake (DMI) and exhibited increased weaning body weight (BW), ADG, and G:F compared to negative controls during a preweaning period (Muya et al., 2017). Additionally, during the postweaning period, calves had increased starter DMI, metabolizable energy intake, final BW, ADG, and G:F (Muya et al., 2017).

No previous research has specifically investigated the effects of *Megasphaera elsdenii* on previously received steers growth performance when transitioned from a low starch receiving diet to an intermediate starch growing diet to determine if administration of the bacterial strain is warranted in the industry. Thus, the objective of this experiment was to evaluate the effects of orally administered *Megasphaera elsdenii* NCIMB 41125 in steers abruptly transitioned from a receiving diet with 4% dietary starch [DM basis] to a growing diet with 38% dietary starch (DM basis).

## Materials and Methods

This research experiment was conducted at the South Dakota State University Ruminant Nutrition Center Feedlot in Brookings, SD, USA. All procedures involving the use of animals in these experiments were approved by the South Dakota State University Institutional Animal Care and Use Committee (Approval #2111-075A).

### Treatments

This experiment used 12 replicate pens per treatment with each pen containing eight steers (*n* = 96 steers/treatment). Each pen was assigned to one of the two treatments in a randomized complete block design (blocked by pen location in feedyard). For both treatments in this trial, steers consumed the same diets prior to and following experiment initiation. Prior to dietary transition, treatments were administered to individual animals and were as follows:

1. Not administered microbial supplement prior to diet change (CON).
2. Orally administered 20 mL microbial supplement ([Lactipro NXT, Axiota Animal Health, Fort Collins, CO] containing 1 × 10^10^ colony forming units *Megasphaera elsdenii* NCIMB 41125) prior to diet change (DFM).

All diets were fortified with vitamins and minerals to exceed nutrient requirements for confinement-fed growing steers (NASEM, 2016). No tylosin phosphate was fed during this experiment and monensin sodium (Rumensin-90; Elanco Animal Health, Greenfield, IN) was fed at 27.5 mg/kg of complete diet (DM basis).

### Cattle Feeding and Management

One hundred and ninety-two Charolais × Angus steers (initial BW shrunk 4% [SBW] = 309 ± 20.6 kg) were sourced from an unrelated 49 d receiving period experiment conducted at the South Dakota State University Ruminant Nutrition Center for use in the current experiment. For the 49 d preceding experiment initiation, all steers consumed a diet (Table 1) based on wheat silage and soybean hulls that contained 4% starch (DM basis). All steers in the current experiment remained in their home pens from the previous receiving experiment to not disturb pen hierarchy and receiving period treatments were equally represented across the current experiment’s treatments.

**Table 1.** Receiving feedlot steers diet containing 4% dietary starch (DM basis)

Item^*^	days −49 to −1
Wheatlage, %	39.62
Dried distillers grains plus solubles, %	9.39
Oat hay, %	10.18
Pelleted soybean hulls, %	35.69
Suspended supplement^†^, %	5.12
Dry matter, %	51.37
Crude protein, %	12.92
Neutral detergent fiber, %	56.49
Acid detergent fiber, %	38.64
Ash, %	6.99
Ether extract, %	2.62
Starch, %	3.97
Neutral detergent fiber from roughage, %	34.03
Net energy for maintenance, Mcal/kg	1.73
Net energy for gain, Mcal/kg	1.04

^*^All values except for dry matter on a dry matter basis.

^†^Suspended supplement contained (DM basis) 36.27% crude protein, 28.00% non-protein nitrogen, 1.63 Mcal/kg of net energy for maintenance, 1.10 Mcal/kg of net energy for gain, 1.62% crude fat, 1.06% crude fiber, 4.62% calcium, 0.43% P, 2.28% K, 0.47% Mg, 5.00% NaCl, 3.38% Na, 0.54% S, 4.00 ppm Co, 200.00 ppm Cu, 20.00 ppm I, 25.15 mg/kg. Of ethylenediamine dihydroiodide, 150.29 ppm Fe, 400.00 ppm Mn, 3.08 ppm Se, 700.00 ppm Zn, 44,092.49 IU/kg of vitamin A, 440.92 IU/kg of vitamin E, and 500.00 g/907 kg of monensin sodium (Rumensin, Elanco, Indianapolis, IN, USA).

On December 08, 2021 steers were processed where an individual BW was recorded and DFM steers were administered treatment. Microbial supplement pouches were rehydrated according to manufacturer specifications and 20 mL of product was orally administered via the manufacturer drenching applicator gun by a trained representative of Axiota Animal Health. Following treatment administration, all steers were immediately transitioned to a growing diet (Table 2) based on high-moisture ear corn and wheat silage that proximately contained 38% starch (DM basis). Each pen was transitioned to the experimental diet at an equivalent DMI as they were consuming the preexperiment diet. The high-moisture ear corn was depleted on day 22 of the experiment, and starting on day 23, high-moisture ear con was replaced by wheat silage and high-moisture corn in the diets maintaining the target concentration of 38% starch and roughage levels. Steers remained in pens until day 49 when steers were weighed, and individual BW was recorded to obtain a final weight for experiment resolution.

**Table 2.** Growing feedlot steers diet containing 38% dietary starch (DM basis)

	Days on feed	
Item^*^	days 1 to 22	days 23 to 49
Wheatlage, %	22.74	31.14
Dried distillers grains plus solubles, %	15.69	20.68
High-moisture ear corn, %	40.70	—
High-moisture corn, %	—	32.73
Dry-rolled corn, %	15.66	10.26
Suspended supplement^†^, %	5.21	5.19
Dry matter, %	51.56	51.50
Crude protein, %	13.83	16.06
Neutral detergent fiber, %	32.01	36.28
Acid detergent fiber, %	19.61	22.96
Ash, %	7.33	8.06
Ether extract, %	2.86	3.15
Starch, %	38.17	34.01
Neutral detergent fiber from roughage, %	18.75	21.38
Net energy for maintenance, Mcal/kg	1.89	1.91
Net energy for gain, Mcal/kg	1.25	1.26

^*^All values except for Dry Matter on a dry matter basis.

^†^Suspended supplement contained (DM basis) 36.27% crude protein, 28.00% non-protein nitrogen, 1.63 Mcal/kg of net energy for maintenance, 1.10 Mcal/kg of net energy for gain, 1.62% crude fat, 1.06% crude fiber, 4.62% calcium, 0.43% P, 2.28% K, 0.47% Mg, 5.00% NaCl, 3.38% Na, 0.54% S, 4.00 ppm Co, 200.00 ppm Cu, 20.00 ppm I, 25.15 mg/kg. Of ethylenediamine dihydroiodide, 150.29 ppm Fe, 400.00 ppm Mn, 3.08 ppm Se, 700.00 ppm Zn, 44,092.49 IU/kg of vitamin A, 440.92 IU/kg of vitamin E, and 500.00 g/907 kg of monensin sodium (Rumensin, Elanco, Indianapolis, IN, USA).

Steers were fed in 58 m^2^ concrete pens with 7.6 m of linear bunk space and equipped with continuous-flow concrete water troughs that were shared between pens within the block. Individual ingredient samples were collected weekly, and DM was calculated following drying in a 60 °C forced air oven until no weight change to calculate DMI. Tabular proximate analysis values for crude protein, neutral detergent fiber (NDF), acid detergent fiber, ether extract, ash, starch, net energy (NE) for maintenance (NE_m_), and NE for gain (NE_g_) were used to build dietary composition tables (NASEM, 2016; Preston, 2016). Steers were fed twice daily at 0800 and 1500 hours and intake was managed according to a slick bunk management system allowing ad libitum access to feed, with minimal day-to-day variation in feed deliveries and carryover feed. Feed was manufactured in a commercial stationary mixer wagon (volume 2.26 m^3^; Roto-Mix 84-8, Roto-Mix LLC, Dodge City, KS) with a scale resolution of 0.45 kg. Weighed feed was delivered to individual pens via a modified chain-driven delivery wagon.

### Rumination and Activity Tracking

Three steers closest to the average initial SBW of each pen (*n* = 72 total) were fitted with an Allflex eSense Flex tag (Allflex Livestock Intelligence; Merck & Co., Rahway, NJ) 7 d prior to experiment initiation to track daily rumination and activity (minutes). Based on previous internal data it was determined via a power analysis that administering three rumination tags per pen was adequate to pick up a 10% difference in rumination between treatments. Tags were placed in the middle one-third of the steer’s right ear and remained in place for the entirety of the experiment. Rumination and activity data were transmitted to a receiver and downloaded into the Allflex Heatime Pro + (Allflex Livestock Intelligence; Merck & Co.) platform and raw data was downloaded for each steer at experiment termination. Data used for analysis began on day −7 at 0000 hours and ended on day 39 at 2400 hours; tag data from days 40 to 49 was excluded from analysis due to system outages that prevented data synching from tag to the Heatime Pro + system. Days −7 to −1 values were averaged and used as baseline rumination and activity measures for statistical analysis.

### Health Management

All steers that were pulled from their home pen for health evaluation were then monitored in individual hospital pens prior to being returned to their home pens. When a steer was moved to a hospital pen the appropriate amount of feed from the home pen was removed and transferred to the hospital pen. If the steer in the hospital returned to their home pen this feed remained credited to the home pen. Health outcomes were characterized as: musculoskeletal (lameness), gastrointestinal (bloat), respiratory (pneumonia), other (pink-eye, etc.), removals (includes animals found dead), and general (dead). One steer from each treatment was treated for respiratory distress; each steer recovered and were returned to their home pens.

### Growth Performance Calculations

Growth performance was calculated on a shrunk live basis. All steers were weighed individually on day 1 processing and on day 49 in a hydraulic squeeze chute mounted on top of load cells (scale readability ± 0.45 kg). Growth performance was based on the initial SBW and day 49 SBW. ADG was calculated as the difference between initial SBW and day 49 SBW divided by days on feed (DOF); G:F was calculated from ADG/DMI.

### Efficiency of Dietary NE Utilization Calculations

Applied energetics measures (observed dietary NE and the ratio of observed-to-expected dietary NE) were assessed for the feeding period. The performance-based dietary NE was calculated from daily energy gain (EG; Mcal/d): EG = (ADG)^1.097^ × 0.0557W^0.75^; where W is the mean equivalent shrunk BW (kg; [NASEM, 2016]) from median feeding SBW (MBW) and AFBW calculated as: (MBW × [478/AFBW], kg; [NASEM, 2016]). From internal historical data from the same source of cattle an AFBW of 606 kg was assumed. Maintenance energy (EM) was calculated by the equation: EM = 0.077 × MBW^0.75^ (Lofgreen and Garrett, 1968). DMI is related to energy requirements and dietary NE for maintenance (NEm; Mcal/kg) according to the following equation: DMI = EG/(0.877NEm—0.41), and can be resolved for estimation of dietary NEm by means of the quadratic formula $x=\frac{-b\pm \sqrt{b^{2}-4ac}}{2a}$, where a = 0.41EM, b = −0.877EM + 0.41DMI + EG, and c = −0.877DMI (Zinn et al., 2008). Dietary NE for gain (NEg) was derived from NEm using the following equation: NEg = 0.877NEm—0.41 (Zinn et al., 2008). Observed-to-expected (O:E) NEm and NEg were a ratio of performance-based dietary NE to tabular dietary NE values (Preston, 2016).

### Statistical Analysis

Growth performance and efficiency of dietary NE utilization were analyzed as a randomized complete block design using the GLIMMIX procedure of SAS 9.4 (SAS Inst. Inc; Cary, NC). Pen was considered the experimental unit, treatment was a fixed effect, and block was a random effect in model analysis. Receiving period treatments were equally distributed across the current experiment’s treatments and initial SBW did not differ between treatments, thus, these variables were not included in the statistical model. Least square means were generated with the LSMEANS option of SAS and significance were determined at *P* ≤ 0.05 and tendencies to differ was observed at 0.05 < *P* ≤ 0.10.

Rumination and activity measures were analyzed as a randomized complete block design using the MIXED procedure of SAS. Individual steer nested within the pen was considered the experimental unit and baseline minutes ruminating and active were used as a covariate in the statistical model. Additionally, treatment, DOF, and their interaction were considered fixed effects in the model and block was included as a random effect. DOF was a repeated measure in the model and a compound symmetry covariance structure was used for model analysis. Least square means were generated with the LSMEANS option of SAS and means were separated and denoted differently (*P* ≤ 0.05) using the pairwise comparison PDIFF option of SAS (SAS Inst. Inc.). Significance was determined at *P* ≤ 0.05 and tendencies were observed at 0.05 < *P* ≤ 0.10.

## Results

The results for growth performance and efficiency of dietary NE are presented in Table 3. No differences (*P* ≥ 0.31) between treatments were observed for final SBW, ADG, DMI, or G:F. Additionally, no difference (*P* = 0.20) was noted for observed NE_m_, observed NE_g_, observed-to-expected NE_m_, and observed-to-expected NE_g_.

**Table 3.** Growth performance and efficiency of dietary net energy (NE) utilization of growing steers transitioned from a 4% starch diet (dry matter basis) to a 38% starch (dry matter basis) and supplemented *Megasphaera elsdenii* NCIMB 41125

	Treatment^*^			
Item	CON	DFM	SEM	*P*-value
Steers, *n*	96	96	—	—
Pens, *n*	12	12	—	—
Growth performance^†^				
Initial shrunk body weight, kg	312	311	2.5	0.62
Final shrunk body weight, kg	379	379	3.1	0.91
Average daily gain, kg	1.36	1.40	0.038	0.56
Dry matter intake, kg	8.22	8.17	0.092	0.69
G:F	0.166	0.171	0.0034	0.31
Efficiency of dietary NE utilization				
Observed NE for maintenance, Mcal/kg	1.74	1.77	0.016	0.20
Observed NE for gain, Mcal/kg	1.11	1.14	0.014	0.20
Observed-to-expected NE for maintenance	0.91	0.93	0.008	0.20
Observed-to-expected NE for gain	0.89	0.91	0.011	0.20

^*^CON steers received no *Megasphaera elsdenii* NCIMB 41125 prior to diet transition and DFM steers received 20 mL of an oral drench (Lactipro NXT, Axiota Animal Health, Fort Collins, CO) containing 1 × 10^10^ colony forming units of *Megasphaera elsdenii* NCIMB 41125 prior to dietary transition.

^†^Growth performance was calculated on a 4% shrunk live basis.

**Figure 1.**
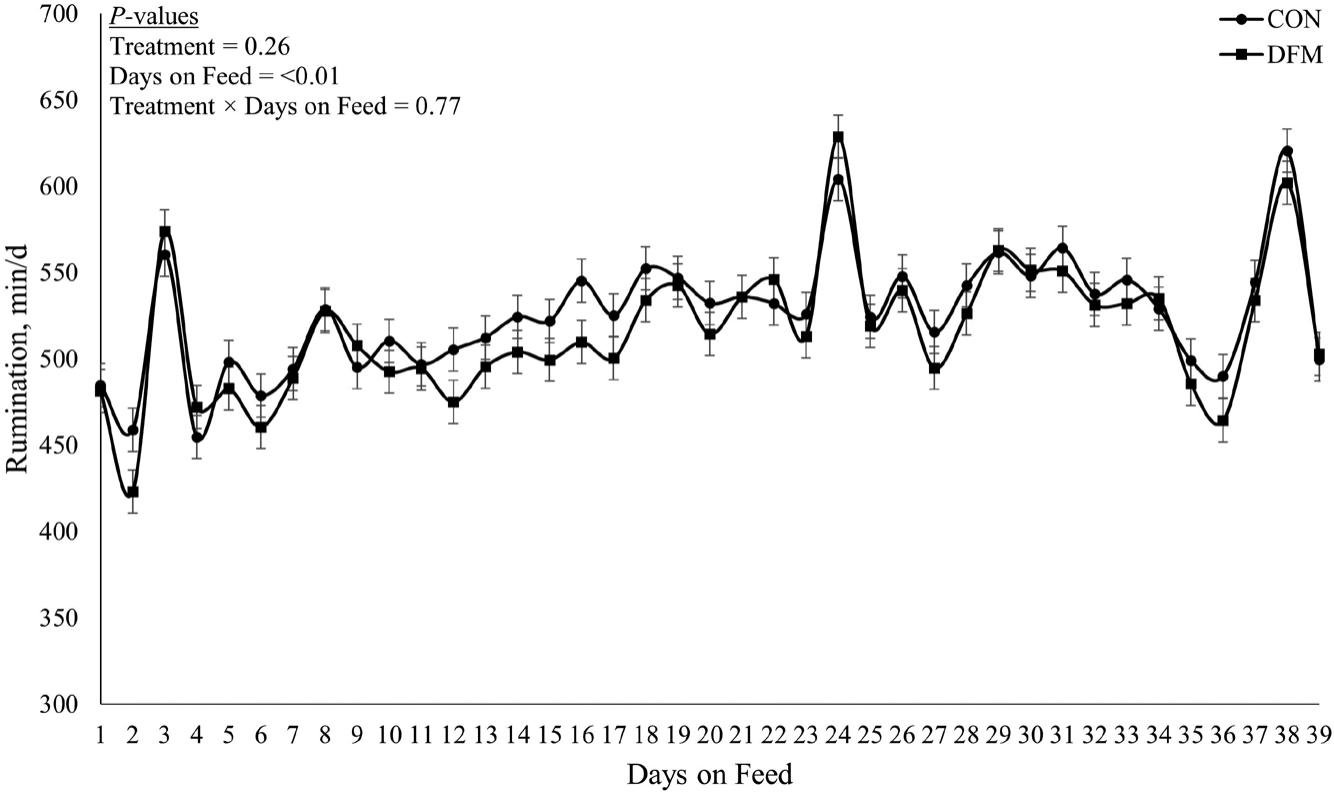
Daily rumination for the first 39 d of growing steers transitioned from a 4% starch diet (dry matter (DM) basis) to a 38% starch (DM basis) and received 0 mL of drench containing *Megasphaera elsdenii* NCIMB 41125 (CON) or received 20 mL of oral drench (Lactipro NXT, Axiota Animal Health, Fort Collins, CO) containing 1 × 10^10^ colony forming units of *Megasphaera elsdenii* NCIMB 41125 (DFM).

**Figure 2.**
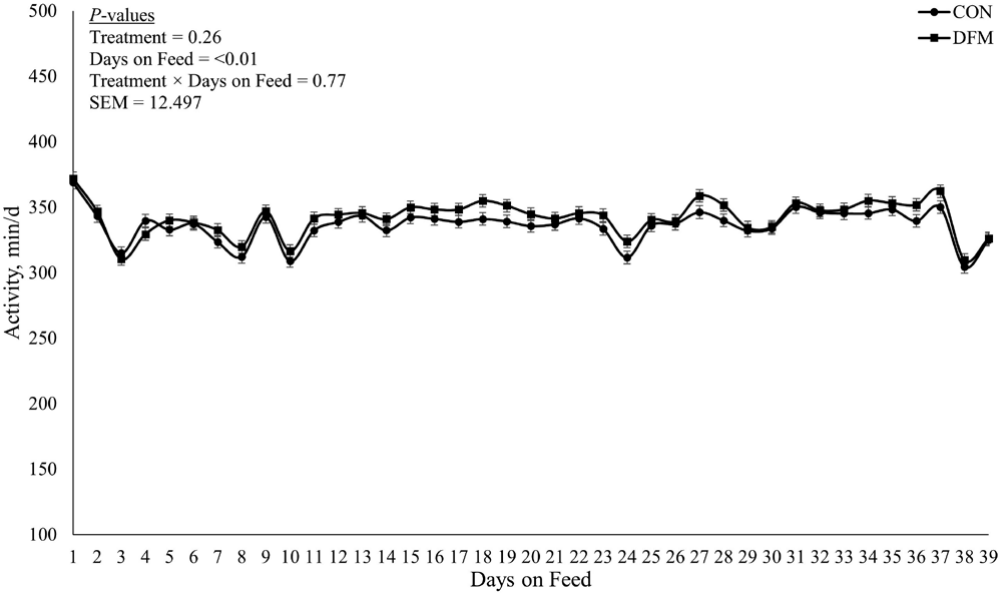
Daily activity for first 39 d of growing steers transitioned from a 4% starch diet (dry matter (DM) basis) to a 38% starch (DM basis) and received 0 mL of drench containing Megasphaera elsdenii NCIMB 41125 (CON) or received 20 mL of oral drench (Lactipro NXT, Axiota Animal Health, Fort Collins, CO) containing 1 × 10^10^ colony forming units of Megasphaera elsdenii NCIMB 41125 (DFM).

Daily rumination and activity results are presented in Figures 1 and 2, respectively. No treatment × day interaction was observed (*P* ≥ 0.74) for daily minutes ruminating or daily minutes active. Additionally, no treatment differences (*P* ≥ 0.13) were observed for minutes ruminating or minutes active. However, a DOF effect (*P* < 0.01) was observed for both minutes of ruminating and minutes active.

## Discussion

For all growth performance, rumination, and activity measures collected, there were no statistical differences between CON and DFM steers. There are a variety of reasons why these responses may have been observed in this experiment including cattle management, dietary roughage level, and monensin sodium supplementation.

As mentioned earlier, the steers used were sourced from a previous experiment conducted at the SDSU RNC and had already been on feed for 49 d prior to experiment initiation. In the previous experiment, steers had been weaned, transported to the feedyard, and processed multiple times where they received vaccinations, a steroidal implant, and visual identification tags. Additionally, these steers had been receiving a mixed ration on a regular feeding schedule, were trained to eat out of a bunk, and were not rerandomized to pens upon the initiation of the current experiment. Consequently, during the initiation of the experiment steers were not subjected to any of the stressors listed above that newly weaned cattle may experience that might lead to erratic feed intake behavior or an off-feed event (NASEM, 2016). In high-risk, newly received steers and bulls, administration of *Megasphaera elsdenii* has been reported to reduce overall mortality and improve growth performance compared to non-dosed controls (Miller et al., 2013). If conditions differed and there were more stressors imposed on the steers, as often occurs in commercial production settings, dosing with DFM may have been a useful tool to aid in the dietary transition imposed in this experiment.

More factors that may have contributed to the lack of responses observed in the current experiment are roughage and roughage NDF levels in the diet. The preexperiment diet contained 50% roughage, 34% roughage NDF, and 4% starch (DM basis) and steers were transitioned to a diet containing 30% roughage, 19% roughage NDF, and 38% starch (DM basis). In the week following experiment initiation, steers consumed 38% less roughage (DM basis) per steer daily and 42% less roughage NDF (DM basis) per steer daily than in the week preceding experiment initiation. With a decrease in roughage intake and an increase in starch intake following experiment initiation it was hypothesized that DFM steers would have improved dietary transition and have improved growth performance. Rumination data from this experiment showed that while roughage concentration of the diet was decreased, minutes spent ruminating remained consistent with their preexperiment baseline levels, likely because of increased DMI. Both the preexperiment and experimental diets are within typical ranges recommended by consulting nutritionists for roughage concentration in receiving feedlot diets (Samuelson et al., 2016). Past literature indicates that ruminal microbiota take 2 to 3 d to adapt to new diets and rumen epithelium may take 5 to 7 d to fully adapt (Brown et al., 2006). Even with the seemingly drastic change in dietary starch and roughage, because steers in this experiment were not further stepped up to a higher concentrate diet, although speculative, based on previous literature (Brown et al., 2006) it is likely that the rumen microbiota and rumen epithelium were able to adapt to the new diets quickly and dosing with DFM had little effect over the 49-d feeding period. *Megasphaera elsdenii* NCIMB 41125 has shown to be most effective in aggressive transition programs where cattle are stepped up to finishing diets quickly by skipping steps or decreasing days on each step (Droulliard et al., 2012; DeClerck et al., 2020). Based on a NASEM (2016) equation that uses forage level in the diet for predicting mean ruminal pH, the mean ruminal pH for steers from both treatments in the current experiment would be approximately 6.01. Additionally, NASEM (2016) recommends adjusting predicted ruminal pH based on management factors that affect the risk for acidosis. Because steers were fed monensin sodium, had consistent feed management, did not transition further to a finishing diet, and had not been on feed for an extended period, the predicted ruminal pH of 6.01 could be increased (NASEM, 2016). Ruminal pH and daily rumination have been investigated in high and low-forage diets previously to characterize how cattle behave when consuming different diets (Chibisa et al., 2016). The growing diet that steers consumed in the current experiment had intermediate starch and forage levels to the high and low-forage diets offered by Chibisa et al. (2016) and predicted ruminal pH and daily minutes ruminating in the current experiment were intermediate to Chibisa et al. (2016). Based on this evidence it is likely that the steers in the current experiment were at a low risk for acidosis and did not benefit from the DFM treatment. In the current experiment, because steers were not fully transitioned to a finishing diet and had adequate roughage to stimulate rumination, it is likely that the buffering effect of rumination was adequate to maintain a pH level in the rumen that allowed for a seamless transition to the experimental diet and DFM was not effective in improving growth performance. In future studies investigating similar dietary transitions, objective measures including clinical depression scores, or fecal consistency scores should be evaluated to determine if DFM affected acidotic conditions following transition until the rumen has time to equilibrate.

Another possible explanation for why DFM was not effective in improving growth performance in the current experiment was that all cattle were supplemented with monensin sodium in both the receiving and experimental growing diets. Previous research has indicated that supplementing monensin sodium can decrease the risk of digestive upset by decreasing instances of erratic feeding behavior and preventing the proliferation of gram-positive lactic acid-utilizing bacteria including *Streptococcus bovis*. In cattle supplemented with monensin, DMI was decreased, indicating that monensin modulates intake and may prevent overeating (Stock et al., 1995). Additionally, individually fed steers supplemented with 27 mg/kg monensin sodium and stepped up to a 100% concentrate ration in 12 d, had decreased variation in DMI across the feeding period compared to control steers (Stock et al., 1995). Reduced intake variation may not always be observed in pen settings because the pen average can mask individual animal variation and pens with fewer cattle likely vary more than pens with larger numbers of cattle (Stock et al., 1995). Because steers in the current experiment were fed in a pen setting, DMI variation was not investigated. However, all steers were across both treatments supplemented with 27.5 mg/kg monensin in the receiving and experimental growing diets. Additionally, Nagaraja et al. (1981) conducted an experiment on ruminally cannulated cows subjected to ground corn-induced acidosis to determine if supplementing monensin 7 d prior to an acidosis event helped maintain pH and DL lactate levels in the rumen. By supplementing monensin 7 d prior to an acidosis challenge, cows had increased rumen pH and decreased levels of D and L lactate until 48 h post-challenge compared to control cows (Nagaraja et al., 1981).

This experiment was conducted during a 49-d period between December 2021 and January 2022. During December 2021 the average temperature was −5 °C (range: −24 to 13 °C) with a minimum wind chill of −38 °C and during January 2022 the average temperature was −11 °C (range: −27 to 6 °C) with a minimum wind chill of −41 °C (South Dakota State University Mesonet, 2024). Thus, during this period it is likely that the steers in the study underwent periods of cold stress. This is evidenced by the mean O:E NEm and NEg values for the experiment being 8% and 10% lower than expected respectively. Alternatively, NE values of diets can be held static and differences can be expressed as changes to the maintenance coefficient (MQ) which is a portion of the EM equation used to predict the maintenance energy requirements of cattle (Zinn et al., 2008). The MQ of cattle in a thermoneutral environment is 0.077 Mcal/BW^0.75^ whereas in the current experiment, the predicted MQ for steers was 0.095 Mcal/BW^0.75^, a 23% increase from thermoneutral conditions. These results are indicative of increased EM due to colder environmental conditions and are consistent with MQ values reported for cattle fed in colder environments (Smerchek and Smith, 2021). Although steers in the experiment may have experienced a certain degree of cold stress during the feeding period, results indicate that administration of DFM had no effect on intake or growth performance.

## Conclusion

Under the conditions of the current experiment, steers immediately transitioned from a diet containing 4% dietary starch (DM basis) to a diet containing 38% dietary starch (DM basis) showed no beneficial effects in growth performance, efficiency of dietary NE utilization, activity, or rumination metrics when dosed with DFM compared to CON. Further research should be conducted to investigate how DFM affects performance metrics and the rumen environment in fed cattle with differing dietary roughage levels, dietary physically effective fiber, monensin supplementation removal, stressors (transit, weaning, feed deprivation, etc.), dietary transition strategies, and health status.

## Data Availability

Data can be made available upon reasonable request to Z.K.S.
